# Global publication productivity and research trends on recurrent ovarian cancer: a bibliometric study

**DOI:** 10.3389/fonc.2024.1422213

**Published:** 2024-07-05

**Authors:** Hua Xu, Lijuan Wang, Dianbo Xu

**Affiliations:** Department of Gynecology, The Affiliated Jiangning Hospital of Nanjing Medical University, Nanjing, China

**Keywords:** ovarian cancer, recurrent, bibliometrics, R language, Bibliometrix, hot topics

## Abstract

**Introduction:**

Recurrent ovarian cancer (ROC) presents a dismal prognosis, persistently devoid of efficacious therapeutic strategies. Over the past decade, significant shifts have transpired in ROC management, marked by the identification of novel therapeutic targets and advancements in biomarker research and innovation. Since bibliometrics is an effective method for revealing scientific literature, we conducted a bibliometric analysis of literature pertaining to ROC. Our exploration encompassed identifying emerging research trends and common patterns, analyzing collaborative networks, and anticipating future directions within this clinical context.

**Methods:**

We conducted a search in the Web of Science Core Collection (WoSCC) to acquire relevant articles as our dataset, which were then exported using R-Studio-2023.12.0–369 software. The Bibliometrix R package was utilized to perform visual analyses on countries, institutions, journals, authors, landmark articles, and keywords within this research field.

**Results:**

A total of 1538 articles and 173 reviews published between 2014 and 2023 were eventually retrieved. The annual growth rate of scientific production was 4.27%. The USA led the way in the number of published works, total citations, and collaboration. Gynecologic Oncology was the most favoured journal in this research field. Vergote I from the University Hospital Leuven, was the most influential author. At last, the most prominent keywords were “chemotherapy” (n = 124), “bevacizumab” (n = 87), and “survival” (n = 65). Clinical outcomes (prognosis, survival), chemotherapy, bevacizumab, and PARP inhibitors (olaparib, niraparib) represented the basic and transversal themes, while antibody-drug conjugate (ADC) and drug resistance were emerging themes. Cytoreduction surgical procedures and tamoxifen were niche themes, while immunotherapy and biomarkers were motor themes and had high centrality.

**Conclusion:**

The trends in the ROC research field over the past decade were revealed through bibliometric analysis. Platinum resistance, ADC, and immunotherapy have emerged as the current prominent research topics.

## 1 Introduction

Ovarian cancer is the third most common gynecologic malignancy worldwide, with its mortality rate also increasing annually (1, 2). The lack of effective screening methods for early-stage ovarian cancer results in 80% of diagnosed cases being in advanced stages, particularly epithelial ovarian cancer (EOC) (3, 4). Cytoreductive surgery followed by platinum-based adjuvant chemotherapy constitutes the cornerstone of initial treatment for ovarian cancer (5, 6). However, recurrence of ovarian cancer appears to be inevitable, with 70% of EOC cases experiencing recurrence within three years postoperatively (7).

Recurrent ovarian cancer (ROC) continues to pose significant challenges for gynecologic oncologists, requiring a delicate balance between limited treatment options and considerations of efficacy and quality of life (8). Particularly for patients with platinum-resistant recurrent ovarian cancer, the lack of potent treatment options often results in poorer prognosis (9).

Over the past decade, significant changes have occurred in the management of ovarian cancer with the discovery of new treatment targets and advances in the study and innovation of biomarkers, such as the identification of the breast cancer BRCA gene and tumors exhibiting homologous recombination deficiency (HRD) (10–12). New treatment modalities in the fields of poly (ADP-ribose) polymerase (PARP) inhibitors, immunotherapy, and hyperthermic intraperitoneal chemotherapy (HIPEC) have the potential to alter the treatment paradigm for ovarian cancer (13–16). These advancements also bring forth more possibilities for the treatment of recurrent ovarian cancer. Therefore, it is particularly important to describe and analyze the current status, progress, and trends in research on ROC worldwide.

Bibliometrics is a tool for studying the production, dissemination, use, and impact of scientific and academic literature (17, 18). It employs mathematical, statistical, and informatics methods to quantitatively analyze scientific literature, revealing information such as the development trends, disciplinary structure, academic collaboration networks, and author influence in a research field (19). It helps researchers understand the characteristics of a particular field of study, predict future research directions, and provide a basis for research decision-making and evaluation. The number of publications on recurrent ovarian cancer has been increasing annually, yet there is currently no bibliometric study. Therefore, we have decided to conduct a bibliometric analysis of research published on ROC in the past 10 years. The aim is to explore emerging trends and common patterns in research on ROC, track collaborations and networks, and anticipate future research directions.

## 2 Methods

### 2.1 Data collection

The Web of Science (WoS) was selected as the data source for this survey. Many scholars believe that the WoS is a premier digital literature resource database that is commonly used for bibliometric analysis (20–22). On January 25, 2024, we searched for related publications in the field of recurrent ovarian cancer through the Web of Science Core Collection (WoSCC) in the Science Citation Index Expanded (SCI-EXPANDED)—1999-present Edition. The search strategy was as follows: TI=((Ovar* NEAR/5 (Cancer* OR Neoplas* OR Carcinom* OR Malignan* OR Tumor* OR Tumour*)) AND ((Recur* OR Recrudesce* OR Relaps*) OR (Platinum and (Refractory OR Resistant OR Sensitive)))) AND PY=(2014–2023). Only articles and reviews which were written in English were included in the analysis. Two researchers independently retrieved and downloaded the literature. In the end, a total of 1365 articles and 173 reviews were filtered out. All the search and download tasks were completed within one day.

### 2.2 Statistical analysis

Bibliographic metadata were downloaded in BibTex format with full record and cited references and exported in R-4.3.2 environment (R-Studio-2023.12.0–369). The Bibliometrix R package was used for the bibliometrics analysis. This package provides various tools and functions specifically designed for conducting bibliometrics analysis (23).

## 3 Result

### 3.1 Overview

**Figure 1:**
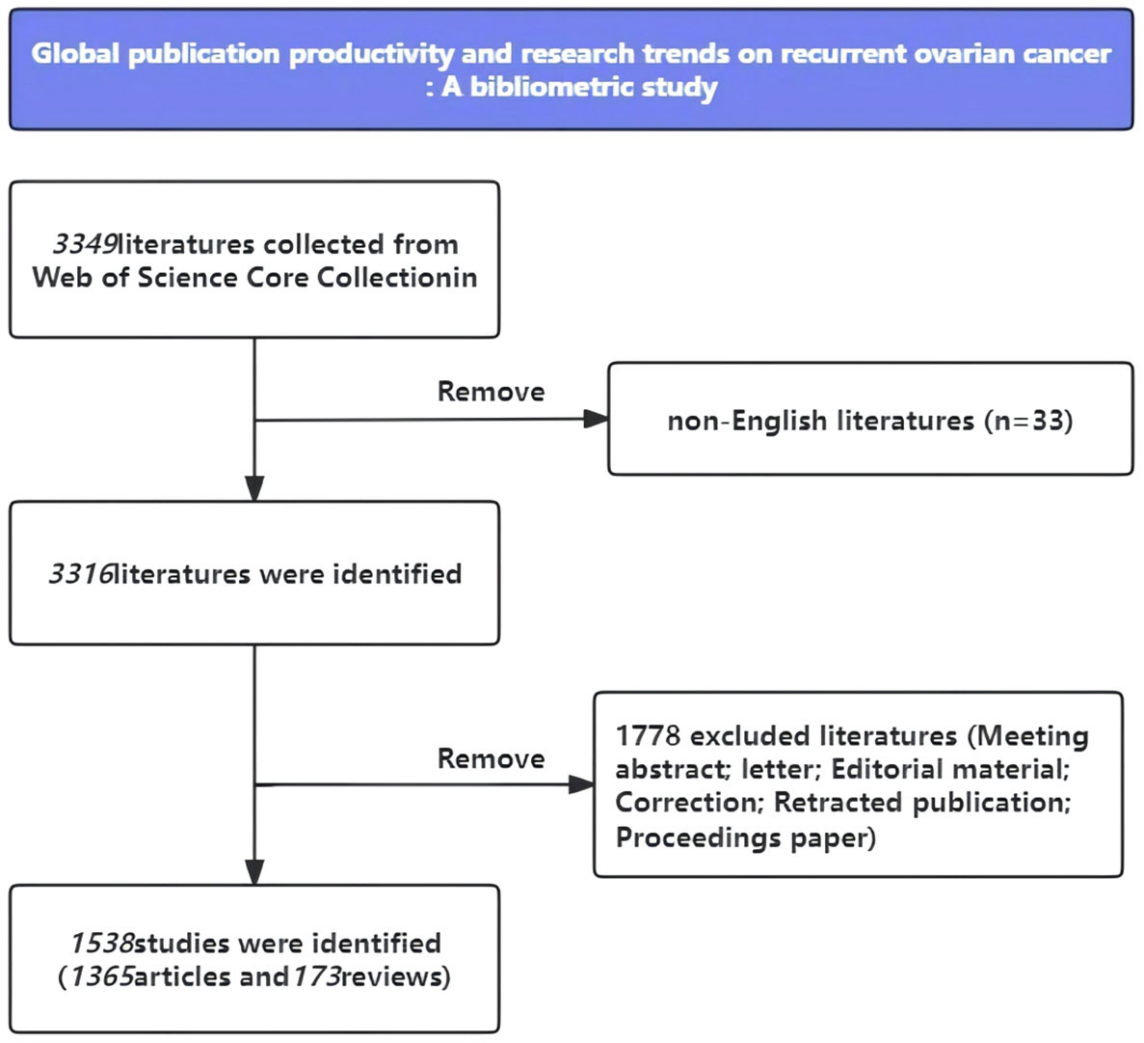
PRISMA flow diagram of the data collection and screening process for the statistic analysis.

**Figure 2:**
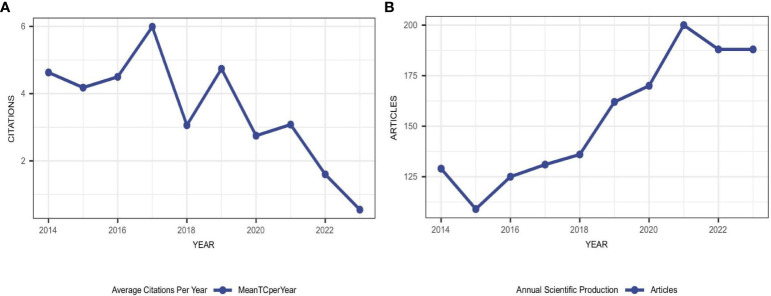
**(A)** Annual publications output and corresponding growth rate between 2014–2023. **(B)** The number of average citations per year in recurrent ovarian cancer.

A total of 3349 documents were collected from WoSCC (as shown in **Figure 1**). We excluded 33 non-English papers and an additional 1778 papers, including letters, meeting abstracts, retracted publications, and proceedings papers. In total, 8,417 authors contributed to 1,538 documents, resulting in an average of 9.03 co-authors per publication. Over the years, there has been a 4.27% annual growth rate in scientific production. This growth is evident from the number of documents, which increased from 129 in 2014 to 188 in 2023 (**Figure 2A**). Furthermore, the mean total citations per year ranged between 1 and 5 citations, with two notable peaks in 2017 and 2019, reaching 5.99 and 4.74 citations per year, respectively (**Figure 2B**). On average, each article received 23.3 citations. The mean total citation per article was highest in 2014, with a mean of 50.94 citations, and lowest in 2023, with a mean of 1.10 citations. These figures reflected the time available for articles to be referenced as citations. Additionally, it is worth mentioning that the international co-authorship percentage reached 24.64%. This indicated a strong collaboration network between different countries and regions in the research field.

### 3.2 Sources

A total of 349 journals published one or more documents in this study. Following Bradford’s law (a bibliometric principle) (24), 10 journals were identified as the core sources responsible for publishing one-third of all the retrieved documents (**Supplementary Figure 1**). **Table 1** presented the top 10 most productive journals, along with their corresponding H-index, total citations, and IF/JCR. The most relevant source, “Gynecologic Oncology”, published a total of 192 articles between 2014 and 2023. It was followed by “International Journal of Gynecological Cancer” (n = 113), “European Journal of Gynaecological Oncology” (n = 39), and “Frontiers in Oncology” (n = 33). Among these, “Gynecologic Oncology” was the most popular journal, with a total of 4375 citations and an H-index of 35. In terms of influence, the most prominent periodical was the “Annals of Oncology” with an impressive IF of 50.5, followed closely by the “Journal of Clinical Oncology” with an IF of 45.4. The “International Journal of Gynecological Cancer” also had a notable impact with an IF of 4.8.

**Table 1:** Top 10 most productive journals in the research field of recurrent ovarian cancer.

Rank	Journal	Publications	H-Index	Total Citations	Publications	IF/JCR (2022)
1	GYNECOLOGIC ONCOLOGY	192	35	4375	192	4.7/Q1
2	INTERNATIONAL JOURNAL OF GYNECOLOGICAL CANCER	113	18	1443	24	4.8/Q1
3	EUROPEAN JOURNAL OF GYNAECOLOGICAL ONCOLOGY	39	3	35	30	0.4/Q4
4	FRONTIERS IN ONCOLOGY	33	6	172	26	4.7/Q2
5	CANCERS	31	8	184	113	5.2/Q2
6	ANNALS OF ONCOLOGY	30	23	2125	25	50.5/Q1
7	JOURNAL OF OVARIAN RESEARCH	29	9	225	15	4.0/Q1
8	ANTICANCER RESEARCH	27	10	260	16	2.0/Q4
8	JOURNAL OF GYNECOLOGIC ONCOLOGY	27	7	233	21	3.9/Q1
10	JOURNAL OF CLINICAL ONCOLOGY	26	20	3105	27	45.4/Q1

IF, Impact Factor; JCR, Journal Citation Reports.

### 3.3 Countries and affiliations

**Figure 3:**
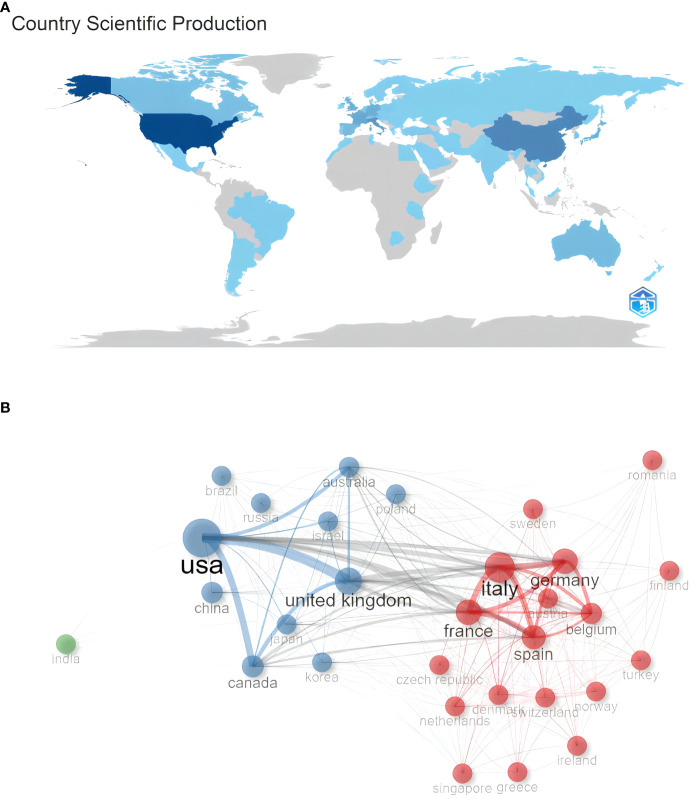
**(A)** Distribution of publications from different countries/regions in recurrent ovarian cancer. **(B)** The collaboration network between countries in recurrent ovarian cancer.

As shown in **Figure 3A**, a total of 65 countries participated in the study of ROC. These countries refer to the locations of corresponding authors, while the depth of the blue color is related to their scientific production: the higher the production, the bluer the color. It was evident that the USA, China, Japan, and several European countries had a deeper color, indicating higher production levels. Furthermore, we presented the top 10 countries with the highest scientific production in **Table 2**. The leading country was the USA with 368 publications, accounting for 23.9% of the total publications. Following closely was China with 284 publications, representing 18.5% of the publications. Italy ranked third with 157 publications, accounting for 10.2% of the total. Notably, China was the only developing country on the list. However, when considering the average article citations, France had the highest average with 58.2 citations, followed by the United Kingdom with 40.5 citations, and Canada with 34.3 citations. In contrast, China, despite having the second-highest number of publications, had a relatively low average of article citations with 8.6 citations.

**Table 2:** Top 10 most productive countries/regions in the research filed of recurrent ovarian cancer.

Rank	Country	Publications	% of 1538 publications	Total Citations	Average Article Citations	MCP/SCP
1	USA	368	23.93%	12596	34.2	0.269
2	CHINA	284	18.47%	2452	8.6	0.081
3	ITALY	157	10.21%	2480	15.8	0.223
4	JAPAN	125	8.13%	1549	12.4	0.048
5	UNITED KINGDOM	77	5.01%	3118	40.50	0.481
6	GERMANY	65	4.23%	1431	22	0.4
7	FRANCE	57	3.71%	3319	58.2	0.439
7	KOREA	57	3.71%	519	9.1	0.123
9	AUSTRALIA	43	2.79%	1188	27.6	0.651
10	CANADA	36	2.34%	1236	34.3	0.389

SCP, Single Country Publications; MCP, Multiple Country Publications.

**Figure 4:**
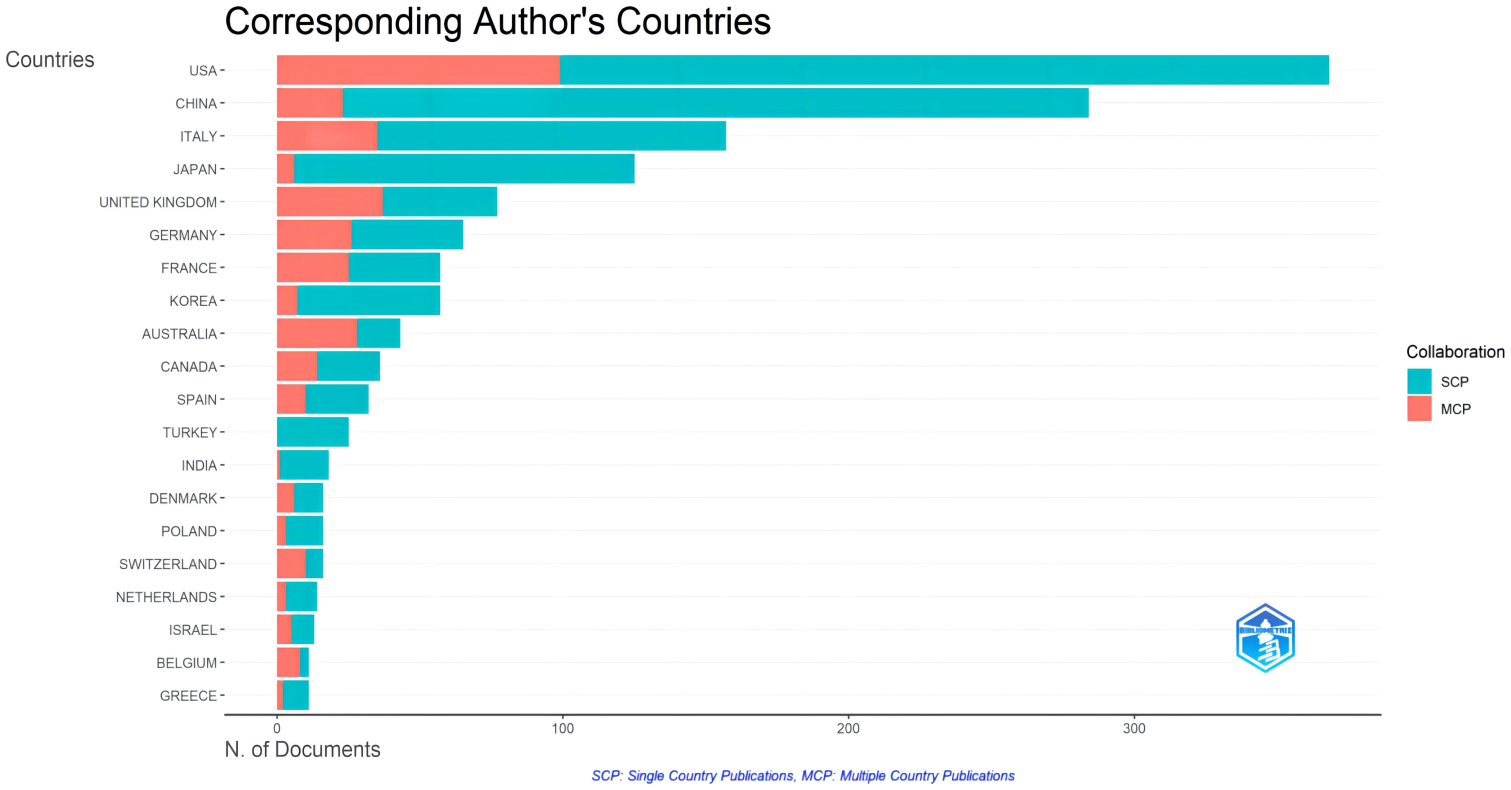
Top 20 countries’ publications partnerships in recurrent ovarian cancer.

Analysis of co-authorship among countries measures the cooperative links based on the number of co-authored documents. **Figure 3B** depicted the collaboration network of the top 30 most productive countries. It revealed that the USA maintains close relationships with other countries, particularly the United Kingdom. Moreover, there was a noticeable trend of mutual cooperation among European countries, with Italy, France, and Germany collaborating closely. **Figure 4** illustrated the publication partnerships in the research field of ROC. The terms “SCP” and “MCP” refer to Single Country Publications and Multiple Country Publications, respectively, indicating the number of papers co-authored by authors of the same or different nationalities. It suggested that international cooperation in this field was quite high, consistent with the findings in **Figure 3B**. However, it should be noted that the number of publications can influence the quantity of MCP. To evaluate the level of collaboration, the MCP ratio (MCP/SCP) was introduced and presented in the last row of **Table 2**. Australia exhibits the highest MCP ratio (0.651), followed by the United Kingdom (0.481) and France (0.439).

In terms of institutions, a total of 2,931 institutions contributed to the retrieved articles. **Table 3** presented the top 10 institutions based on the number of publications. The University of Texas MD Anderson Cancer Center ranked first with 126 articles, followed by Memorial Sloan Kettering Cancer Center and Fudan University with 123 and 99 articles respectively. **Supplementary Figure 2** illustrates the cumulative publications of the top five institutions over a span of 10 years. Notably, Fudan University initiated its study on recurrent ovarian cancer in 2017 and has consistently maintained a high level of scientific output since then.

**Table 3:** Top 10 most productive institutions in the research field of recurrent ovarian cancer.

Rank	Affiliation	Country	Publications	% of 1538 publications
1	UNIV TEXAS MD ANDERSON CANC CTR	USA	126	8.19%
2	MEM SLOAN KETTERING CANC CTR	USA	123	7.99%
3	FUDAN UNIV	CHINA	99	6.43%
4	DANA FARBER CANC INST	USA	82	5.33%
5	UNIV CATTOLICA SACRO CUORE	ITALY	78	5.07%
6	UNIV OKLAHOMA	USA	71	4.62%
7	UNIV SYDNEY	AUSTRALIA	68	4.42%
8	OHIO STATE UNIV	USA	67	4.36%
9	UNIV MILANO BICOCCA	ITALY	64	4.16%
10	SEOUL NATL UNIV	KOREA	55	3.58%

### 3.4 Authors

**Table 4** presented the top 10 authors with the highest productivity, where SEHOULI J had the highest number of publications with 50 documents. VERGOTE I and SCAMBIA G followed closely with 49 and 47 documents, respectively. Meanwhile, VERGOTE I received the highest total citations among the ten authors, with a total of 9,662 citations. According to Hirsch, the H-index is defined as: “A scientist has index h if h of his or her Np papers have at least H citations each and the other (Np – H) papers have ≤H citations each.” (25). The G-index addresses the limitations of the H-index by considering citation scores for evaluation (26). Generally, the H-index can be used to assess the quantity and impact of a researcher’s scholarly output, while the G-index places greater emphasis on highly cited articles. VERGOTE I (H-index=27), COLOMBO N (H-index=24), and OZA AM (H-index=21) were the top three authors ranked by H-index. Meanwhile, VERGOTE I (G-index=49), SCAMBIA G (G-index=48), and COLOMBO N (G-index=46) had the highest G-index, which was consistent with the results ranked by total citations. Considering the variations in career lengths, the M-index serves as a correction for temporal clues to help identify truly successful researchers (27). By dividing the H-index by the number of years, we obtained a new index called the M-index. Only three authors had an M-index over 2, with the highest M-index belonging to VERGOTE I with 2.5.

**Table 4:** Top 10 most productive authors in the research field of recurrent ovarian cancer.

Rank	Author	Publications	Total Citations	H_index	G_index	M_index
1	SEHOULI J	50	1969	20	44	1.8
2	VERGOTE I	49	7153	27	49	2.5
3	SCAMBIA G	48	2326	21	48	1.9
4	COLOMBO N	46	5204	24	46	2.2
5	WANG Y	39	794	15	27	1.4
6	COLEMAN RL	38	3352	17	38	4.5
7	OZA AM	34	5081	21	34	1.9
8	LORUSSO D	33	1433	16	33	1.6
9	MOORE KN	31	1310	19	31	2.1
9	RAY-COQUARD I	31	3999	17	31	1.5

**Figure 5:**
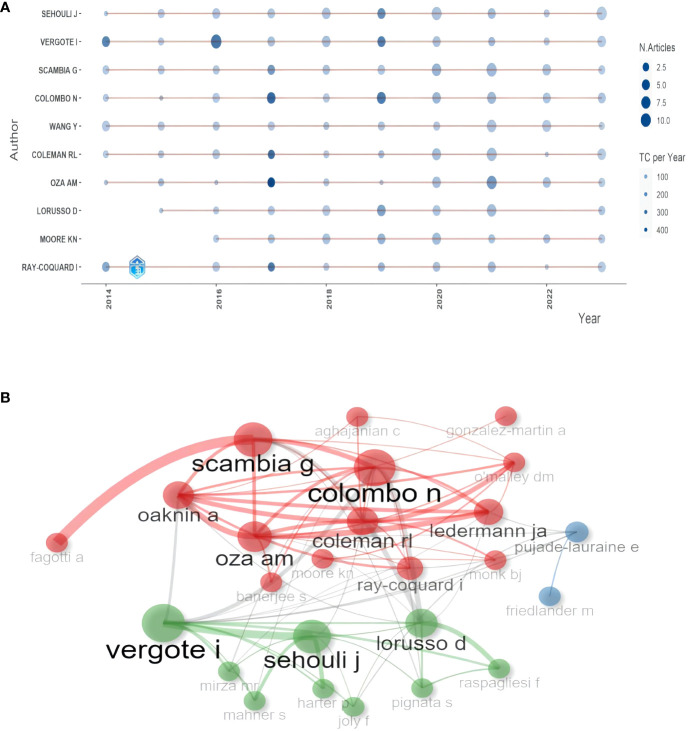
**(A)** Top 10 authors’ production over time. **(B)** The collaboration network of authors.

In order to investigate the temporal variation of productivity, we used Bibliometrix to obtain a timeline view of the top ten authors with the highest productivity. As shown in **Figure 5A**, the node size represented the number of articles, and the shade of color represented the total citations. It was evident that most authors consistently published articles during the period of 2014–2023. **Figure 5B** showed the collaboration network among the top 30 productive authors. The size of the colored blocks represented the number of publications, while the thickness of the lines connecting authors reflected the number of co-authored articles between them. It was obvious that VERGOTE I had the highest degree of collaboration with other authors and SCAMBIA G worked most closely with FAGOTTI A.

### 3.5 Articles and references

Researchers employ citations to track the evolution of concepts over time and identify papers from a vast array of publications that may hold the greatest value for their research (28). Furthermore, the Local Citation Score (LCS) refers to the count of document citations within a specific dataset. Evidently, articles that receive extensive citations can offer comprehensive insights into scientific progress (29). Publications were ranked in descending order based on their LCS to identify the most valuable articles, as presented in **Table 5**, which presented the top 10 most local cited articles, comprising 8 original articles and 2 reviews. These articles were primarily published between 2014 and 2019. The article ranked first is “Bevacizumab Combined With Chemotherapy for Platinum-Resistant Recurrent Ovarian Cancer: The AURELIA Open-Label Randomized Phase III Trial.” It was published in the Journal of Clinical Oncology in 2014 with 300 local citations.

**Table 5:** The top 10 articles with the most local citation scores in the research field of recurrent ovarian cancer.

Rank	Title	First Author	Journal	Year	DOI	LC	GC	LC/GC Ratio (%)
1	Bevacizumab Combined With Chemotherapy for Platinum Resistant Recurrent Ovarian Cancer: The AURELIA Open Label Randomized Phase III Trial	PUJADE-LAURAINE E	Journal of Clinical Oncology	2014	10.1200/JCO.2013.51.4489	300	1077	27.86
2	Olaparib tablets as maintenance therapy in patients with platinum-sensitive, relapsed ovarian cancer and a BRCA1/2 mutation (SOLO2/ENGOT-Ov21): a double-blind, randomized, placebo-controlled, phase 3 trial	PUJADE-LAURAINE E	Lancet Oncology	2017	10.1016/S1470–2045(17)30469–2	151	1170	12.91
3	Rucaparib maintenance treatment for recurrent ovarian carcinoma after response to platinum therapy (ARIEL3): a randomized, double-blind, placebo-controlled, phase 3 trial	COLEMAN RL	Lancet	2017	10.1016/S0140–6736(17)32440–6	129	1077	11.98
4	Bevacizumab and paclitaxel–carboplatin chemotherapy and secondary cytoreduction in recurrent, platinum-sensitive ovarian cancer (NRG Oncology/Gynecologic Oncology Group study GOG-0213): a multicenter, open-label, randomized, phase 3 trial	COLEMAN RL	Lancet Oncology	2017	10.1016/S1470–2045(17)30279–6	116	393	29.52
5	Olaparib maintenance therapy in patients with platinum sensitive relapsed serous ovarian cancer: a preplanned retrospective analysis of outcomes by BRCA status in a randomized phase 2 trial	LEDERMANN J	Lancet Oncology	2014	10.1016/S1470–2045(14)70228–1	108	1069	10.10
6	Fifth Ovarian Cancer Consensus Conference of the Gynecologic Cancer InterGroup: recurrent disease	WILSON MK	Annals of Oncology	2017	10.1093/annonc/mdw663	77	171	45.03
7	Final overall survival and safety analysis of OCEANS,a phase 3 trial of chemotherapy with or without bevacizumab in patients with platinum-sensitive recurrent ovarian cancer	AGHAJANIAN C	Gynecologic Oncology	2015	10.1016/j.ygyno.2015.08.004	70	214	32.71
8	“Platinum resistant” ovarian cancer: What is it, who to treat and how to measure benefit?	DAVIS A	Gynecologic Oncology	2014	10.1016/j.ygyno.2014.02.038	58	285	20.35
9	Antitumor activity and safety of pembrolizumab in patients with advanced recurrent ovarian cancer: results from the phase II KEYNOTE-100 study	MATULONIS UA	Annals of Oncology	2019	10.1093/annonc/mdz135	57	379	15.04
10	Safety and Antitumor Activity of Anti–PD-1 Antibody, Nivolumab, in Patients With Platinum-Resistant Ovarian Cancer	HAMANISHI J	Journal of Clinical Oncology	2015	10.1200/JCO.2015.62.3397	53	789	6.72

LC, Local Citations; GC, Global Citations.

In comparison to LCS, the Local Citation Reference (LCR) allows for rapid identification of the most relevant articles in a research field. **Table 6** presented the top 10 most local cited references.

**Table 6:** Top 10 most locally cited references in the research field of recurrent ovarian cancer.

Rank	Title	First Author, Year	Journal	LCR
1	Bevacizumab Combined With Chemotherapy for Platinum Resistant Recurrent Ovarian Cancer: The AURELIA Open Label Randomized Phase III Trial	PUJADE-LAURAINE E, 2014	J CLIN ONCOL	300
2	OCEANS: a randomized, double-blind, placebo-controlled phase III trial of chemotherapy with or without bevacizumab in patients with platinum-sensitive recurrent epithelial ovarian, primary peritoneal, or fallopian tube cancer	AGHAJANIAN C, 2012	J CLIN ONCOL	211
3	New response evaluation criteria in solid tumors: revised RECIST guideline (version 1.1)	EISENHAUER EA, 2009	EUR J CANCER	207
4	Niraparib Maintenance Therapy in Platinum-Sensitive, Recurrent Ovarian Cancer	MIRZA MR, 2016	NEW ENGL J MED	165
5	Olaparib tablets as maintenance therapy in patients with platinum-sensitive, relapsed ovarian cancer and a BRCA1/2 mutation (SOLO2/ENGOT-Ov21): a double-blind, randomized, placebo-controlled, phase 3 trial	PUJADE-LAURAINE E, 2017	LANCET ONCOL	151
6	Incorporation of bevacizumab in the primary treatment of ovarian cancer	BURGER RA, 2011	NEW ENGL J MED	141
7	Olaparib maintenance therapy in platinum-sensitive relapsed ovarian cancer	LEDERMANN J, 2012	NEW ENGL J MED	131
8	Rucaparib maintenance treatment for recurrent ovarian carcinoma after response to platinum therapy (ARIEL3): a randomized, double-blind, placebo-controlled, phase 3 trial	COLEMAN RL, 2017	LANCET	129
9	Cancer Statistics, 2016	SIEGEL RL, 2017	CA-CANCER J CLIN	129
10	A phase 3 trial of bevacizumab in ovarian cancer	PERREN TJ, 2011	NEW ENGL J MED	121

LCR, local cited references.

### 3.6 Keywords

**Figure 6:**
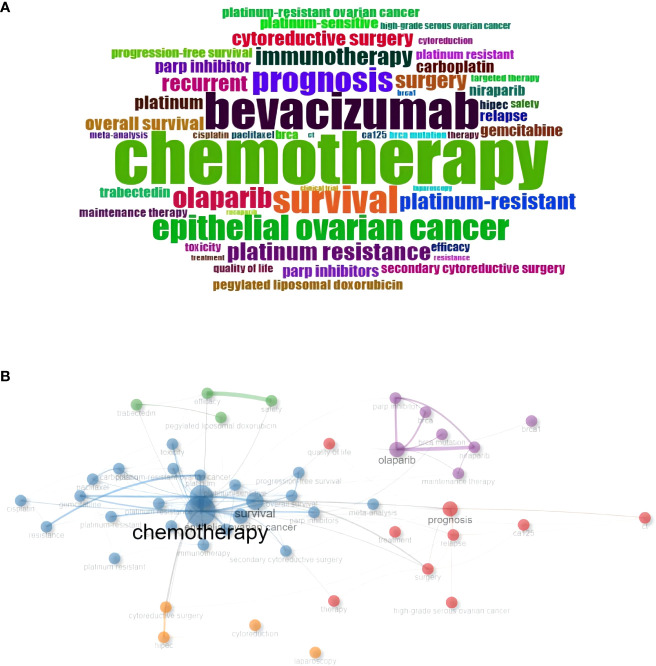
**(A)** Keyword cloud of the retrieved articles. **(B)** Co-occurrence analysis of keywords.

Keyword analysis is a process that enables us to accurately identify the hot topics and trends in a specific field (30). Bibliometrix was employed for visualizing the occurrence and frequency of keywords. This study encompassed a total of 2597 keywords proposed by the authors in the articles. After excluding search terms and synonymous keywords, the keyword “chemotherapy” had the highest frequency, occurring 124 times. It was followed by “bevacizumab” with 87 occurrences and “survival” with 65 occurrences. **Figure 6A** presented the WordCloud after removing the search terms, where the font sizes were proportional to the frequencies of the keywords. In a research paper, When two keywords appeared together in an article, they were referred to as “co-occurrence keywords” and generally had some correlation, which could be expressed through the co-occurrence frequency. The distance between nodes in the network depended on their co-occurrence frequency. **Figure 6B** displayed a co-occurrence network of the top 50 keywords, and the size of each node represented the total number of co-occurrence occurrences.

**Figure 7:**
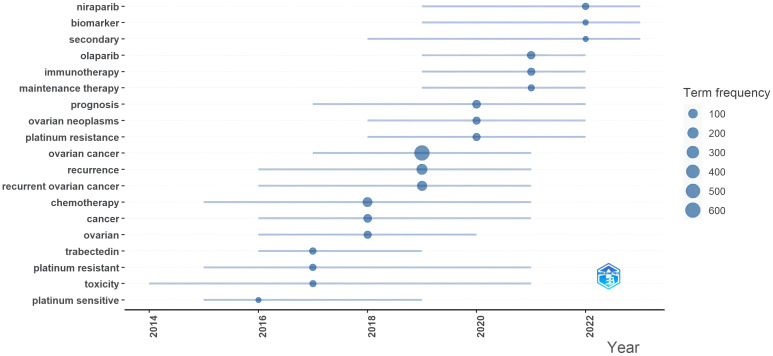
Trend topics from 2014–2023 in the research field of recurrent ovarian cancer.

The trend topics were displayed in **Figure 7**, based on the top three keywords that appeared at least 10 times each year. The size of the nodes indicated the frequency of term appearances, while the gray lines represented the duration. Notably, nilarpari and biomarkers made their initial appearance in 2019 and experienced a sudden surge in 2022. Other emerging keywords in the past three years included olaparib, immunotherapy, maintenance therapy, and platinum resistance.

### 3.7 Themes and thematic evolution

We applied a clustering algorithm to the keyword network and generated a thematic map. The thematic map consisted of mainstream themes, including isolated topics (niche themes), new topics (emerging themes), hot topics (motor themes), and essential topics (basic themes). Each bubble represented a network cluster, with the bubble name determined by the keywords with the highest occurrence value. The size of the bubble was proportional to the occurrence frequency of the cluster’s keywords, and its position was determined based on cluster centrality and density. In the thematic evolution map, the cutting point was fixed at 2019, with different clusters indicated by color coding. The Walktrap algorithm was employed to cluster the data in this study.

**Figure 8:**
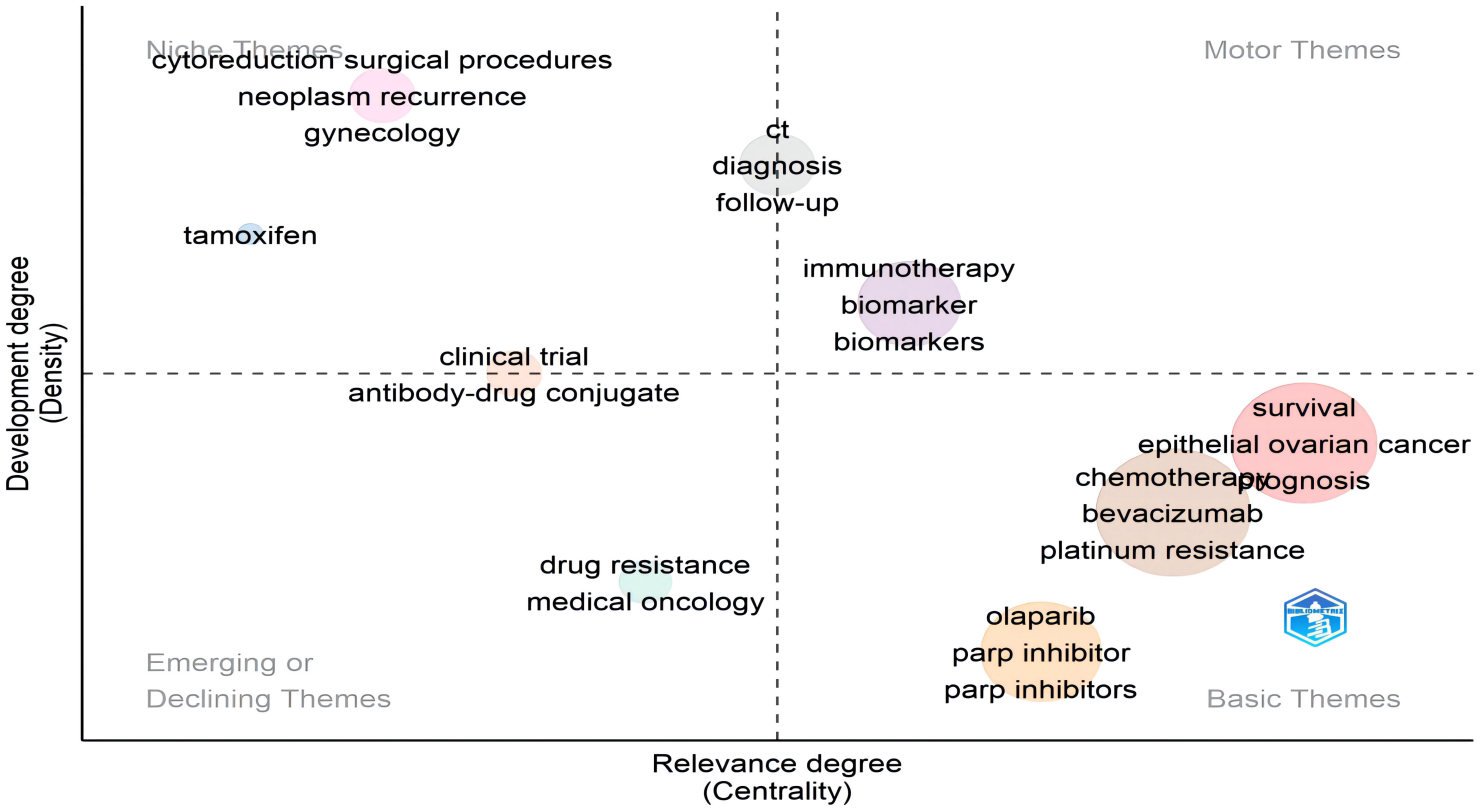
Thematic map.

**Figure 9:**
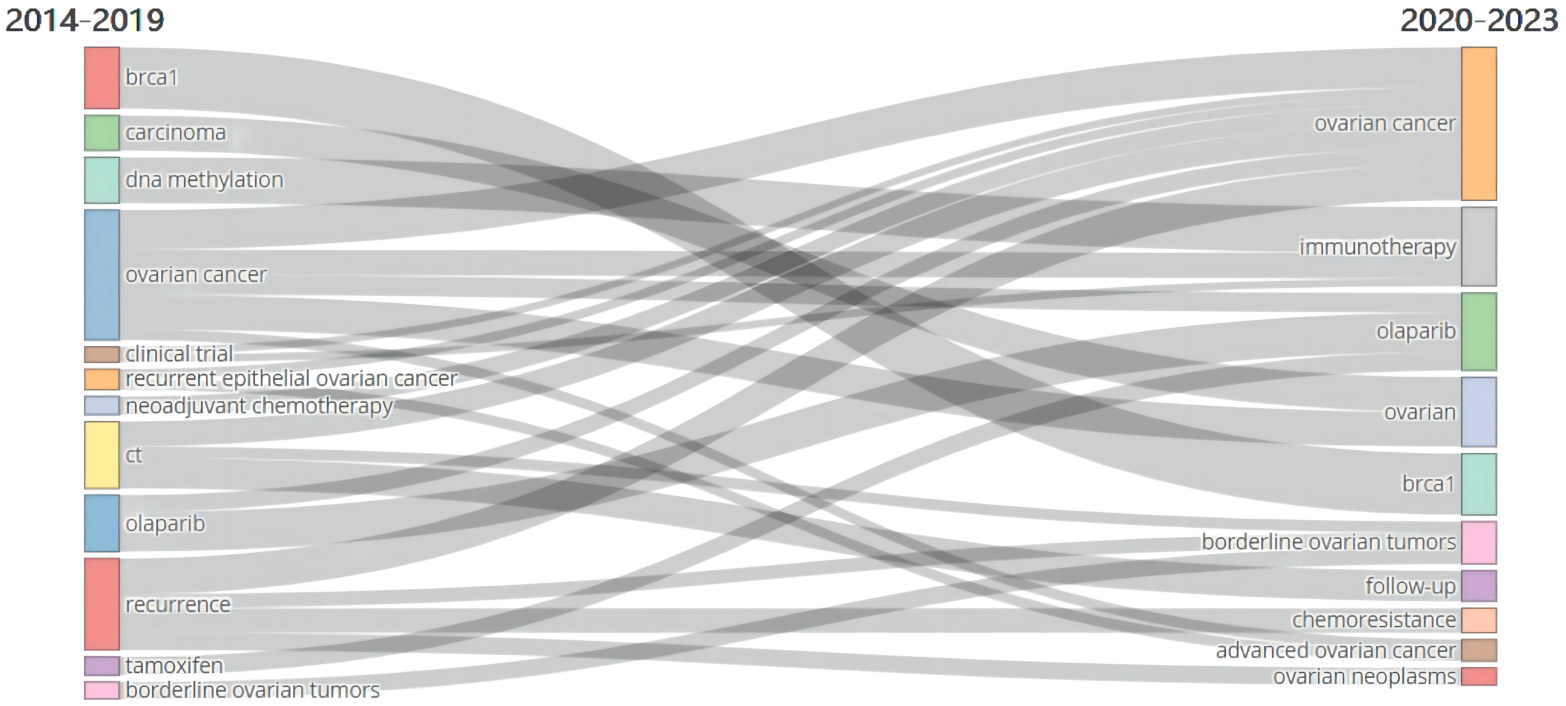
Thematic evolution.

The main themes and trends were shown in **Figures 8** and **9**. The thematic map (**Figure 8**) indicated that immunotherapy and biomarkers were hot topics, while antibody-drug conjugate and drug resistance were emerging themes. Clinical outcomes (prognosis, survival), chemotherapy, bevacizumab, and PARP inhibitors (olaparib, niraparib) represented the basic and transversal themes. Cytoreduction surgical procedures and tamoxifen were isolated topics. **Figure 9** illustrates the major thematic areas and their evolution over two different time periods: 2013 to 2019 and 2019 to 2023, along with their relationships. It primarily demonstrated the transition from “clinical trials,” “neoadjuvant chemotherapy,” “olaparib,” and “recurrence” to “ovarian cancer,” while “DNA methylation” and “ovarian cancer” shifted towards “immunotherapy.”

## 4 Discussion

We conducted a scientometric analysis of 1,538 publications related to ROC over the past decade. During this period, there was a steady increase in the overall quantity of scientific output. The production of articles grew at an average annual rate of 4.27% and reached its peak in 2021, with only slight negative growth rates observed in 2015 and 2022. Furthermore, the years 2019 and 2021 exhibited relatively high growth rates, indicating a significant surge in research interest in the field. The upward trend indicated that the research field was flourishing. Moreover, the high average citations per article implied that publishing research on recurrent ovarian cancer in reputable journals poses no challenge. **Figure 2B** suggested that publications in 2017 might have exerted a significant influence on the study of recurrent ovarian cancer. Over the past decade, the mean total citations per article generally declined. While the number of citations is an important metric for assessing academic impact, it is important to acknowledge the influence of time. Earlier studies tended to have a higher likelihood of being cited frequently compared to more recently published ones. Therefore, the academic impact of earlier studies does not necessarily surpass that of later ones (22, 31).

The analysis of countries/regions revealed a widespread distribution of scientific publications across all continents, which reflected the global prevalence of ROC. The leading hubs for scientific production in this field are North America (USA and Canada), Europe (Italy, UK, France, Germany), and Asia (China, Japan, Korea, India). Notably, the United States stood out as the leading country in scientific productions and citations, with 368 papers and a total citations of 12,596. Based on the data provided, it could be firmly concluded that the US was at the forefront of research in this field. Interestingly, China, as the only developing country on the list, demonstrated significant progress in the study of ROC among developing nations. However, China’s average article citations of 8.6 were relatively low. This might be attributed to a focus on quantity rather than quality among Chinese academics (32). In general, research on ROC has been predominantly concentrated in developed countries like the USA. Therefore, the inclusion of China in this list was encouraging for developing countries. It indicated that China has made notable strides in the study of ROC despite the lower average citation count. In the collaboration network, US engaged in collaborations with most countries, and its collaboration with the United Kingdom was particularly close. Additionally, European countries had established extensive collaborations among themselves. According to the institutions’ analysis, the University of Texas MD Anderson Cancer Center ranked first among the top 10 institutions in terms of the number of articles. The list included five institutions from the USA, which was consistent with the countries’ analysis.

The analysis of journals revealed that “Gynecologic Oncology” received the highest number of citations and publications, suggesting that it was the most widely read and popular journal within the field of gynecologic oncology. On the other hand, “Annals of Oncology” ranked first in terms of Impact Factor (IF), indicating that it had the highest average number of citations per published article in the research of ROC. Furthermore, based on Bradford’s Law, a methodology used to determine the core journals in a specific field, a compilation of 10 core journals was presented in **Supplementary Figure 1**. These core journals were identified as the main contributors to the important publications within the field, indicating that they held significant influence and authority in the research community. The analysis of journals in this manner could be beneficial for researchers as it could provide them with valuable guidance in selecting the most appropriate journals to target for their publications. By understanding the popularity, IF, and core journal rankings, researchers can ensure that their work reaches the right audience and has the potential to make a significant impact in the field of gynecologic oncology research.

In the authors’ analysis, the number and citations of publications were used to evaluate individual authors. SEHOULI J emerged as the leader in terms of the total number of publications. VERGOTE I had the highest H-index and M-index, followed by COLOMBO N. Additionally, VERGOTE I had the highest G-index, followed by SCAMBIA G. It is recommended to focus on VERGOTE I, COLOMBO N, and SCAMBIA G to keep up with significant advances in the field of ROC. Furthermore, based on **Figure 5A**, it was evident that these ten prolific authors had dedicated a substantial amount of time to this field and continue to produce articles. This suggests that there are still valuable perspectives to explore in the study of ROC, indicating ongoing research opportunities from another standpoint.

In the articles’ analysis, we employed bibliometrix to identify articles with the highest LCS - indicating the most frequently cited articles. Among the top 10 most cited articles, clinical trials dominated, suggesting that the treatment of ROC is still in an exploratory stage. Specifically, Pujade-Lauraine et al. conducted a study in 2014 and found that in patients with platinum-resistant recurrent ovarian cancer, the use of bevacizumab in combination with chemotherapy significantly improved the patients’ progression-free survival (PFS). Their research findings indicated that the hazard ratio for PFS events in patients receiving bevacizumab combined with chemotherapy was 0.48, with a median PFS of 6.7 months, compared to a median PFS of 3.4 months in the chemotherapy-alone group (33). Also in 2014, a study by Jonathan Ledermann et al. found that in platinum-sensitive recurrent ovarian cancer patients with BRCA mutations, the median PFS was 11.2 months for the olaparib group and 4.3 months for the placebo group. It suggested that olaparib might offer greater benefits for patients with BRCA mutations, providing new insights into the personalized treatment of ROC (34).

SOLO2/ENGOT-Ov21 is a double-blind, randomized, placebo-controlled, multicenter, Phase III clinical trial that compared the efficacy of olaparib tablets as maintenance therapy in platinum-sensitive recurrent ovarian cancer patients with BRCA1/2 mutations. Patients were randomly assigned to two groups, with one receiving olaparib tablets and the other receiving placebo, in a ratio of 2:1. The primary endpoint was PFS assessed by the investigators. The results showed a median PFS of 19.1 months in the olaparib group, significantly longer than the placebo group’s 5.5 months (35). In 2017, the clinical trial ARIEL3 found that maintenance therapy with rucaparib significantly prolonged PFS, particularly in patients with BRCA mutations or homologous recombination deficiencies (HRD). The results showed a median PFS of 16.6 months in the rucaparib group compared to 5.4 months in the placebo group for patients with BRCA mutations. In patients with HRD, the median PFS was 13.6 months in the rucaparib group and 5.4 months in the placebo group (36). ARIEL3 provided further evidence that use of a poly(ADP-ribose) polymerase inhibitor in the maintenance treatment setting versus placebo could be considered a new standard of care for women with platinum-sensitive ovarian cancer following a complete or partial response to second-line or later platinum-based chemotherapy. In 2015, Junzo Hamanishi et al. investigated the safety and anti-tumor activity of the anti-PD-1 antibody nivolumab in patients with platinum-resistant ovarian cancer. The study included 20 patients who received intravenous nivolumab treatment, with 80% experiencing side effects, but severe adverse events accounted for only 2%. The best overall response was 15%, including two patients achieving durable complete response. The median PFS was 3.5 months, and the median overall survival (OS) was 20 months. Their study provided evidence for further large-scale trials of anti-PD-1 antibody therapy in ovarian cancer (37).

In 2019, Ursula A. Matulonis et al. conducted a study on the application of pembrolizumab in advanced ROC. The study aimed to evaluate the treatment response of patients to pembrolizumab, a novel therapeutic approach. The study included two cohorts of patients: cohort A received one to three prior lines of treatment with a platinum-free interval (PFI) or treatment-free interval (TFI) between 3 and 12 months, and cohort B received four to six prior lines with a PFI/TFI of ≥3 months. Pembrolizumab was given intravenously every three weeks until disease progression, toxicity, or completion of two years. The primary endpoints were objective response rate (ORR) and PD-L1 expression measured as combined positive score (CPS). Secondary endpoints included duration of response (DOR), disease control rate (DCR), PFS, OS, and safety. The results demonstrated an ORR of 7.4% in cohort A and 9.9% in cohort B, with a median DOR of 8.2 months and not reached, respectively. Additionally, analysis based on PD-L1 expression levels identified ORRs of 4.1% for CPS <1, 5.7% for CPS ≥1, and 10.0% for CPS ≥ 10 (38). This trial indicated a relatively low ORR but a higher DCR with pembrolizumab treatment in ROC. Moreover, patients with a CPS ≥10 exhibited a higher ORR, which could potentially guide future research directions.

Keywords form the essence of a paper summary, as they provide a glimpse into the article’s topic. By analyzing the keywords cloud, it was observed that among the 1,538 articles, excluding the entry terms, “chemotherapy,” “bevacizumab,” and “survival” were the frequently occurring keywords. **Figure 7** revealed that chemotherapy and platinum resistance remained prominent subjects, whereas in the past three years, there had been a remarkable surge in research focused on immunotherapy and maintenance therapy, driven by the findings of several clinical trials (33–41). Olaparib and niraparib had emerged as prominent subjects in the management of ROC (39). From 2014 to 2023, themes related to treatment modalities, surgery, and prognosis remained unchanged, encompassing chemotherapy, bevacizumab, and PARP inhibitors. These treatment modalities have consistently played crucial roles in ovarian cancer management, with continuous research in these areas.

Platinum resistance had emerged as a central theme in recent years in ROC, as patients with platinum resistance often have poorer prognoses (42, 43). Hence, “clinical trials” and “antibody-drug conjugates(ADC)” were emerging trends that were likely to occupy a significant position in scientific literature soon (44, 45). ADC has emerged as a fresh form of anticancer therapy. Binding particular antigens on the membrane of tumor cells facilitates deactivation of downstream cancer-causing pathways, thereby provoking immune responses (46). In comparison to conventional chemotherapeutic medications, coupling an antigen-targeting antibody with a cytotoxic medicine enables selective drug delivery to cells expressing the antigen, augmenting effectiveness and minimizing overall toxicity. An ADC consists of a tumor-specific antibody joined with a cytotoxic substance through a molecular linker and will ultimately be internalized and discharged into tumor cells (47). Numerous tumor antigens are differentially expressed in OC cells and can be utilized for this innovative therapeutic strategy. Folate receptor alpha (FRa) is expressed in over 80% of serous ovarian malignant tumors and exhibits no response to chemotherapy (48). Mirvetuximab soravtansine (MIRV) is an ADC specifically designed to target FRa, with its efficacy demonstrated in multiple clinical trials (49–51). As additional therapeutic targets are identified, ADC may potentially offer patients more effective treatment options.

The study’s main limitation is associated with its intrinsic nature. Bibliometric analysis produces quantitative information that may lead to over- or underestimation. Inadequate standardization of items, such as affiliations, can introduce bias into the data. The study’s limited focus only on documents within the WOS database undermines its ability to reflect the complete global research activity on ROC. Additionally, quantitative data, especially the number of citations, may be influenced by the duration for which the publication can accrue references. Furthermore, multiple studies have been updated and published multiple times, reporting on diverse endpoints and various follow-up periods.

## 5 Conclusion

Research on ROC has been conducted for several decades. The bibliometric study analyzed articles related to ROC between 2014 and 2023, systematically exploring the annual growth trends in publications, journals, countries, institutions, authors, landmark articles, collaborative networks, and keyword analysis. Over the past decade, the focus of research on ROC has shifted from chemotherapy to molecular targeted drugs and immunotherapy. By combining article and keyword analysis, platinum resistance, ADC, and immunotherapy have emerged as the current prominent research topics. Therefore, future research will primarily concentrate on conducting clinical trials of immunotherapy drugs. Additionally, there is an urgent need to explore more sensitive and effective biomarkers to guide targeted therapy effectively.

## Data availability statement

The original contributions presented in the study are included in the article/**Supplementary Material**. Further inquiries can be directed to the corresponding author.

## Author contributions

HX: Writing – review & editing, Writing – original draft, Investigation, Funding acquisition, Formal analysis, Data curation, Conceptualization. LW: Writing – review & editing, Writing – original draft, Investigation, Funding acquisition, Formal analysis, Data curation, Conceptualization. DX: Writing – review & editing, Supervision, Software, Methodology, Investigation, Funding acquisition, Formal analysis, Conceptualization.
