# Supplementary material for: Global publication productivity and research trends on recurrent ovarian cancer: a bibliometric study
Source: Front Oncol. 2024 Jul 5;14:1422213. doi: 10.3389/fonc.2024.1422213 (PMC11257877; doi:10.3389/fonc.2024.1422213)
Supplement: Supplementary file 1 [file DataSheet_1.docx]

Supplementary Material

**
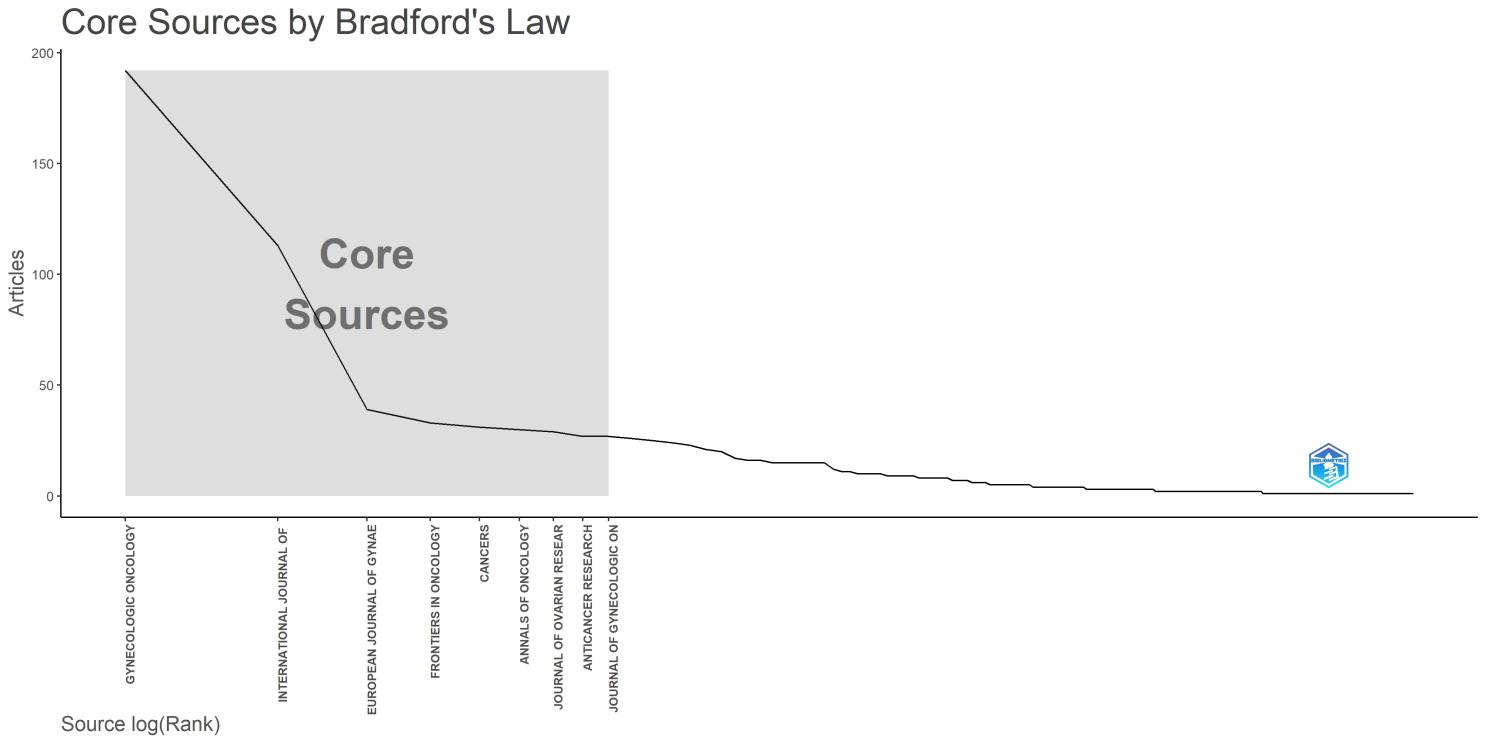
**

**Supplementary Figure 1.** Source clustering through Bradford’s law


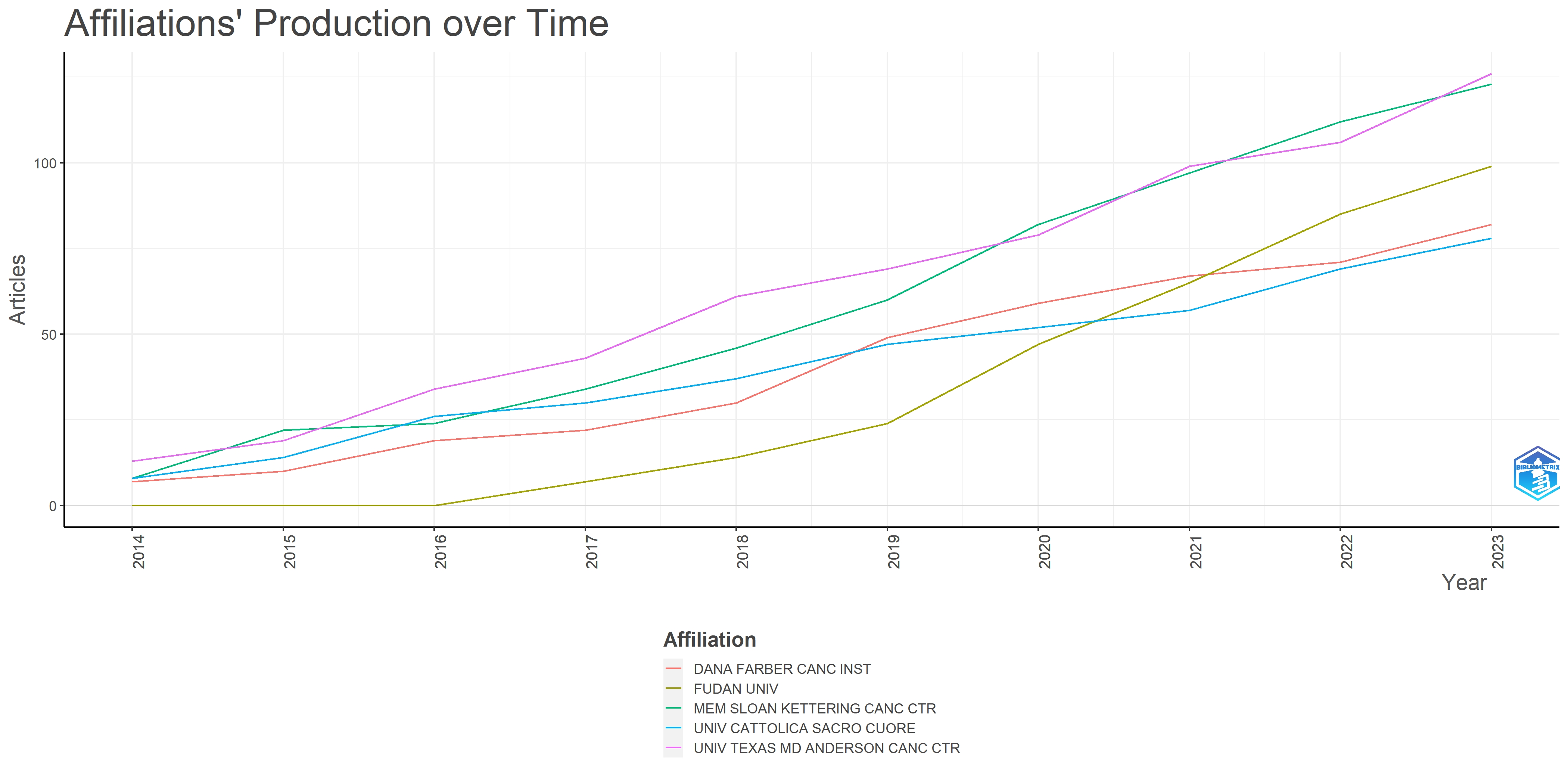
**Supplementary Figure 2.** Growth trend of the top 5 institutions
